# Adolescent binge drinking causes sex-specific deficits in behavioral reactivity, endocrine recovery from stress and altered expression of hypothalamic KCC2 in males

**DOI:** 10.1007/s00213-025-06969-7

**Published:** 2025-12-08

**Authors:** Bilge Büyükdemirtaş, Milan Benn, Han Bin Kwon, Ellie Rosen, Laverne Camille Melón

**Affiliations:** 1 Department of Biology, Middletown, CT 06459 USA; 2 Program in Neuroscience and Behavior, Wesleyan University, Middletown, CT 06459 USA; 3 College of Integrative Sciences, Wesleyan University, Middletown, CT 06459 USA

**Keywords:** Adolescent social drinking, Sex difference, KCC2, Slc12a5, Hypothalamus, Social defeat stress

## Abstract

**Rationale:**

Binge drinking during adolescence is associated with a higher risk of developing Alcohol Use Disorders and impaired stress reactivity in adulthood. The hypothalamic-pituitary-adrenal axis matures during adolescence and is under strict GABAergic control. Yet, how early binge drinking alters GABAergic signaling in this region, and whether these changes contribute to later-life disruptions in stress reactivity, remain unknown.

**Objectives:**

Our goal with these set of experiments was to test the hypothesis that adolescent binge drinking would impair maturation of GABAergic signaling in the paraventricular nucleus (PVN) and lead to aberrant behavioral and neuroendocrine stress reactivity.

**Methods:**

Adolescent male and female C57BL/6J mice were given access to 20% ethanol or tap water under Drinking in the Dark-Multiple Scheduled Access paradigm from PD28 to PD42 and went through abstinence until adulthood. At adulthood (PD60+), the behavioral and endocrine response to two stressors, forced swim stress and social defeat stress, were evaluated.

**Results:**

Only females with a history of adolescent drinking showed abnormal behavioral reactivity to stress, with increased immobility in the forced swim stressor task. Females with a history of adolescent binge drinking displayed a hypo-corticosterone response after social stress and males showed delayed negative feedback to the forced swim stressor. PVN-enriched tissue from these mice showed no changes in transcription of *Gabrg2*, but males showed a significant increase in expression of *Slc12a5*, which encodes for the chloride potassium co-transporter, KCC2. These males showed no change in KCC2 protein when evaluated by immunohistochemistry.

**Conclusions:**

Taken together, these data show that adolescent binge drinking in pair-housed mice is associated with male-specific potential vulnerability in hypothalamic inhibitory signaling and disrupted adult stress reactivity in a sex- and stressor- dependent manner.

## Introduction

Over 8% of adolescents and young adults (ages 12–20) in the United States were past month binge drinkers (SAMHSA OR Substance Abuse and Mental Health Services Administration 2024). For adolescents, binge drinking, or intake that increases blood ethanol levels to ≥ 0.08 g/dL is defined as 3 drinks in a 2 h period for boys aged 9–13 and girls aged 9–17 (Donovan 2009). This pattern of risky drinking during adolescence is associated with an increased rate of diagnosis of alcohol use disorder (Patrick et al. 2023)and an increased rate of diagnosis of stress-associated pathologies, including anxiety and PTSD (Stein et al. 2017)in adulthood. Exposure to alcohol during this developmental stage is associated with sex-dependent changes in hypothalamic neuropeptide signaling (Towner et al. 2022), hypothalamic-pituitary-adrenal (HPA) axis reactivity (Logrip et al. 2013)and stress or anxiety-related behavior. Adult women report higher stress, anxiety, and depression co-morbidity with alcohol use than men, indicating a sex difference in the relationship between alcohol and negative affect (Peltier et al. 2019). These studies suggest that binge drinking during adolescence leads to sex-dependent aberrant stress response and reactivity in adulthood. While the relationship between risky drinking and dysregulated stress reactivity is noted regardless of developmental stage, the mechanisms that drive this relationship may be different for adolescents.

Adolescence, overlapping with pubertal maturity, is a crucial time for the development of the hypothalamus and connectivity of the neuroendocrine HPA stress axis (Green and McCormick 2016; Romeo 2017; Romeo et al. 2006). Puberty marks a shift in sex associated changes in HPA reactivity; adult women display a greater HPA axis response to an infusion of corticotropin releasing hormone (CRH) compared to men (Panagiotakopoulos and Neigh 2014)whereas pre-pubescent girls display a lower HPA axis response to CRH infusion when compared to pre-pubescent boys (Hollanders et al. 2017). Indeed, one of the hallmarks of pubertal maturation of the HPA axis is an increase in activity; girls show more pronounced activity of the axis by age 13 (Gunnar et al. 2009; Stroud et al. 2011). A shift in HPA axis activity is also observed in rodent models during adolescence. In rats, pituitary reactivity to stress matures by early adolescence, and adrenal corticotropes establish an adult-like profile during late adolescence (Romeo 2017; Burke and Miczek 2014). Prior to this, prepubertal male and female rats show a prolonged corticosterone (CORT) response to stressors, with a higher peak CORT concentration compared with adults following stress (Burke and Miczek 2014; Klein and Romeo 2013).

Contrary to what is seen in clinical studies, preclinical studies support a disproportionate impact of adolescent binge drinking on stress reactivity in males. For example, adolescent binge drinking has been shown to predispose male but not female rats to stress-induced anxiety-like behaviors in adulthood (Healey et al. 2023). A history of adolescent binge drinking in rats is associated with increased social anxiety (Marsland et al. 2023), novelty-induced anxiety, atypical behavior in the forced swim stress task as well as atypical glucocorticoid levels and CRH immunoreactivity in the paraventricular nucleus (PVN) (Brancato 2021). Early adolescent alcohol reduces social investigation and preference in only male rats but reduces open arm measures in both sexes (Towner and Varlinskaya 2020; Varlinskaya et al. 2014). Male rats with an adolescent alcohol history show a hypoactive CORT response to an ethanol challenge in adulthood, as well as reduced*Avp*mRNA expression in the PVN (Allen et al. 2016, 2011). Adolescent alcohol exposure is associated with reduced exploration of open arm of the elevated plus maze (EPM) in males (Matthews et al. 2023)and increased baseline plasma CORT levels (Sánchez-Marín et al. 2022). Male and female mice with a history of adolescent alcohol also exhibit increased anxiety-like behaviors in the open field maze, the EPM, and in novel object exploration tests in adulthood; as well as increased depression-like behaviors in forced swim stress (Van Hees et al. 2022). These findings suggest that the impact of adolescent binge drinking on stress reactivity in adulthood is sex-dependent, yet the mechanisms driving these differences remain unknown.

The HPA axis is under strict GABAergic control, as half the inputs CRF neurons receive are GABAergic (Miklós and Kovács 2002). One of the conditions that is necessary for GABA to exert its inhibitory effect is establishment of the Cl^−^electrochemical gradient. One of the transporters that is necessary for this electrochemical gradient is potassium-chloride co-transporter KCC2 (Rivera et al. 2004). In the hypothalamus, the activation when faced with a stressor requires removal of inhibitory constraint which is achieved by rapid internalization of KCC2 from the cell membrane (Sarkar et al. 2011; Ye et al. 2012). As a potent GABAergic regulator, alcohol changes function and expression of genes related to GABA signaling, including receptors (Melón et al. 2017, 2019), packaging proteins (Eravci et al. 2000), and Cl^−^balance proteins such as KCC2 and NKCC1 (Melón and Maguire 2016). Therefore, our study aimed in part to uncover the molecular point of impact adolescent alcohol has on the GABAergic signaling system that regulate the HPA axis. The goal of the present work was to test the hypothesis that the impact of adolescent binge drinking on stress reactivity in adulthood is sex and stress-modality dependent. Adolescent male and female C57BL/6J mice in a pair-housed intermittent binge drinking paradigm were used to replicate and further characterize the findings in the field that one sex may be particularly vulnerable to the effects of early drinking on stress reactivity in adulthood. Adult behavioral and neuroendocrine reactivity to a non-social stressor (forced swim) and social stressor (encounter with a larger, trained aggressor) were evaluated alongside transcriptional changes in genes whose protein products reflect stability of presynaptic and synaptic GABAergic signaling. We found a history of adolescent binge drinking leads to deficits in behavioral and endocrine reactivity to stressors in adulthood that was qualitatively different across sex, with males showing unique upregulation of KCC2 in the hypothalamus and a sluggish endocrine recovery to social stress.

## Methods

### Timeline

**Fig. 1:**
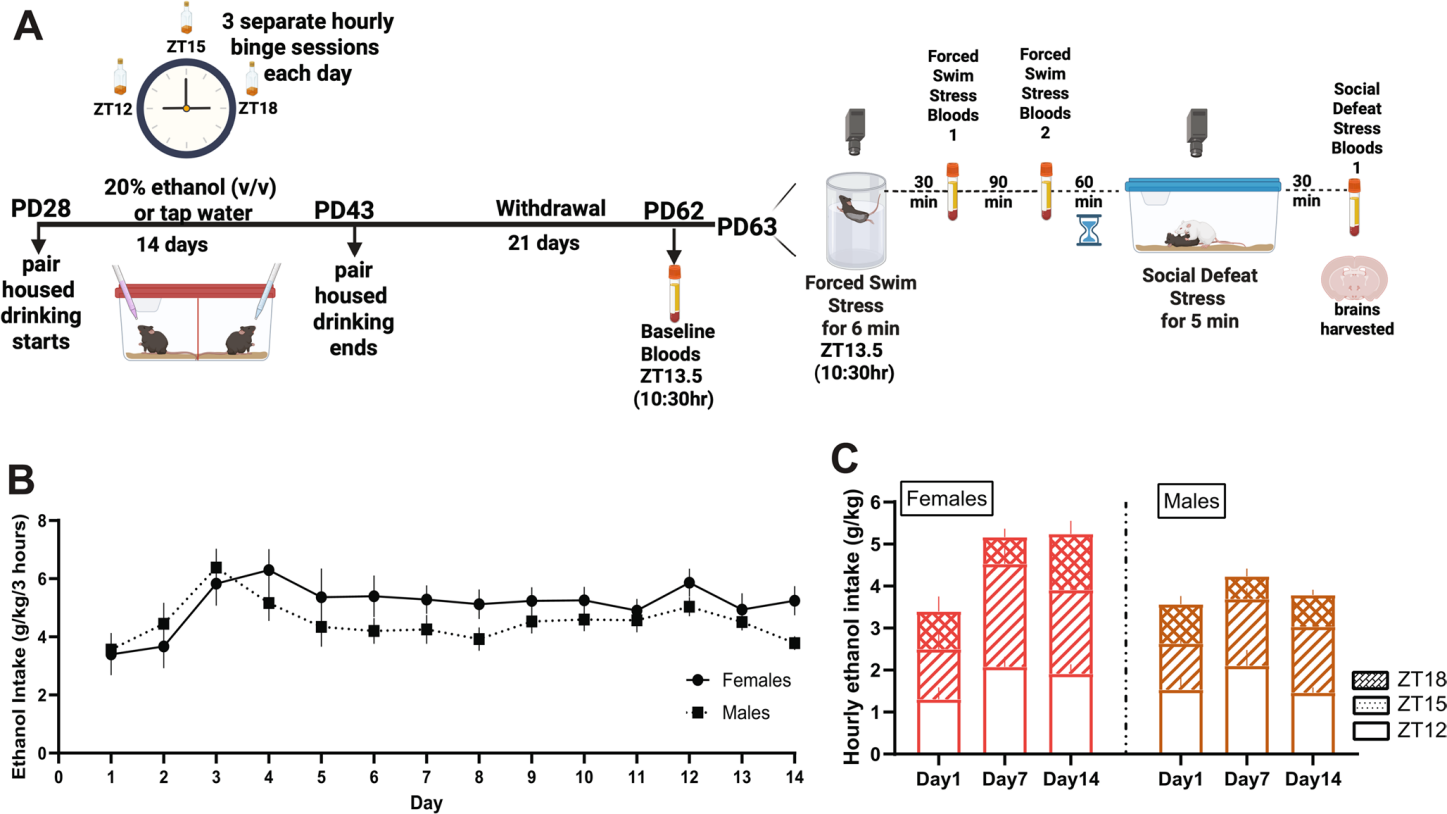
Adolescent pair-housed male and female mice consume similar amounts of alcohol in a limited access paradigm. **A**. Experimental timeline. **B** Daily drinking values for males and females across 14 days was compared using a repeated measures ANOVA which supported similar intakes and no escalation of drinking over time for males and females in the pair-housed binge drinking paradigm. C. Fraction of daily drinking at each session on days 1, 7, and 14 for males was stable over time with mice drinking least during ZT18. Panel A was created in BioRender. Melon, L. (2025) https://BioRender.com/k73m559

Animals were co-housed with a same-sex littermate separated across a perforated divider and given access to a 20% (v/v) ethanol solution or tap water three times a day starting on PD28. Beginning at the start of the dark cycle, each one-hour binge session spaced by two hours of ad libitum water access. The drinking lasted for 14 days and ended on PD43. Mice were then allowed to go through withdrawal until they reached adulthood. On PD62, baseline bloods were taken from all mice, and on PD63 mice underwent forced swim stress at 10:30am. Stress reactivity bloods were taken 30 minutes after forced swim stress, and stress recovery bloods were taken at 120 minutes after forced swim stress. After an additional 1-hour recovery period, or 3 hours after forced swim stress, mice underwent social defeat stress with a sex-matched trained CFW aggressor. Thirty minutes after social defeat stress mice were euthanized, their brains and trunk bloods were collected for further analysis (Fig. 1A).

### Animals

#### Experimental animals

C57BL/6J mice were bred in-house at the Wesleyan University animal facility from breeders sourced from Jackson Laboratories (#00064). A total of 6 cohorts of littermates from timed pregnancies were used across experiments. Mice were maintained on a 14:10 light/dark cycle until weaning (PD21). At weaning, mice were moved to a reversed 12:12 light/dark (09:00 h lights off) reverse cycle room and co-housed in standard shoebox cages with their littermates until PD25 when they were separated into sex-matched pairs. Pairs were separated with a red translucent perforated divider to ensure mice sensory cues across partners; this divider was in place throughout the experiment. Mice were maintained in ICV housing in a temperature- (68–72 F) and humidity- (35–40%) controlled room. Experiments were performed according to guidelines and procedures accepted by Wesleyan University’s Institutional Animal Care and Use Committee.

#### Aggressor animals

Retired breeder Swiss Webster (CFW) mice were used as aggressors in the social defeat stress experiments. Females were ovariectomized while males were kept intact to ensure territorial aggression. Mice were purchased from Charles River Laboratories and were kept in same sex group-housing at 14:10 light/dark cycle for 2 weeks, until females’ wounds have healed. After that, the CFW females and males were paired, and housed in 14:10 light/dark cycle for 3 weeks to ensure bonding was achieved between sexes.

#### Drinking in the dark multiple scheduled access (DID-MSA)

At PD28, mice received limited access to ethanol (20%, v/v in tap water) in graduated drinking tubes (modified serological pipettes with double-balled sippers) in three-hourly drinking bouts, as previously described (Melón et al. 2013). Each hourly access period was separated by two hours of water-only access, when regular bottles were returned. The first session of alcohol access began at ZT12 (09:00 h, lights out). Alcohol access sessions were continued for 14 days until PD42. Controls were identically treated except graduated drinking tubes contained water. Mice were tested three weeks after the last drinking session, as adults (PD60+).

#### Blood corticosterone levels

All blood samples were collected from the submandibular vein except for the last time point which was taken from the trunk following decapitation. Blood was collected into K2EDTA tubes (Becton Dickinson, #044939). Baseline blood was taken the day before testing (PD62). Following forced swim stress, bloods were collected at 30-minute (stress reactivity) and 120-minute (stress recovery) timepoints. Following social defeat stress, blood was collected at a 30-minute timepoint. Samples were centrifuged for five minutes at 12,000G and the serum stored in −20 C. For free corticosterone determination, an ELISA assay was performed following manufacturer protocol (Enzo Life Sciences, #ADI900-097). Samples were run in duplicate, with an on-plate standard curve of known corticosterone concentrations and absorbance read at 450 nm on a multireader spectrophotometer (Beckman Coulter Spectramax, DTX880). For the forced swim stress analysis two statistical outliers were removed from the water drinking males and one male, and two female statistical outliers were removed from the ethanol-exposed group.

#### Forced swim stress

On experiment day (ZT13.5; 10:30 h), mice were placed in a deep round beaker filled with 2 L of tap water (23–25 C) and allowed to swim for six minutes. The session was recorded by a ceiling mounted camera. Mice were immediately returned to home cages following the stress test. Their behavioral response to the stressor was coded using BORIS software (Friard and Gamba 2016) by two independent and blinded scorers. Two parameters were measured in two mutually exclusive classes of behaviors, swimming (mobile) and floating (immobile). The latency to the initial bout of immobility and the total time mice spent floating in the final 4 min of the test were evaluated. When paws were visible, paw movements were taken as the ultimate measure of swimming vs. floating. If paws were invisible, the ripples in the water were taken as an indicator of paddling therefore were considered as swimming. Lack of ripples in the water were taken as the measure of floating behavior.

#### Social defeat stress

Three hours after the end of the forced swim stress (ZT16.5; 13:35 h), experimental mice were introduced for five minutes into the home cage of a trained sex-matched CFW aggressor using the Miczek paradigm as previously described (Newman et al. 2019). In following prior work establishing social defeat stress in both males (Miczek et al. 1982, 2022)and females (Newman et al. 2019), the stress task took place in the homecage of the CFW aggressors. Prior to the stress task, food, water, hut, nesting material, and the partner were removed from the homecage of the aggressor into a spare cage. The experimental mouse was introduced into the homecage of the aggressor, and the cage was closed by a clear plexiglass to avoid mice escaping the set up. Mice were allowed to interact for 5 min, at the end of which the experimental mouse was removed from the aggressor’s cage and returned to its own homecage. Partner and cage contents were returned to the homecage of the aggressor. Each aggressor faced 3–5 experimental mouse a day depending on the size of the cohort. The protocol was the same for both sexes. The sessions were video recorded by a ceiling mounted camera. Two blinded scorers used BORIS software to code three classes of behaviors; defense posture (experimental mice were standing on their hind legs), avoidance (experimental mice were running away from the aggressor), and neutral (experimental mice were performing exploratory activities). The percentage of time spent in defense and avoidance postures were evaluated across all groups.

#### Brain harvest, hypothalamus dissection, and cryosectioning

Thirty minutes after the end of the social defeat stressor, mice were anesthetized with isoflurane, sacrificed by guillotine-assisted decapitation, and brains harvested.

For qPCR, brains were rapidly removed and sliced using an ice-cold 1 mm brain matrix. Slices were kept in RNAlater (Thermo Fisher, #AM7040) overnight until tissue harvests. PVN was grossly microdissected and placed in 1mL of Trizol homogenization buffer for homogenization by handheld homogenizer.

For immunohistochemistry, brains were placed into 4% paraformaldehyde. Graded sucrose (10% and 30%) incubations were used prior to freezing in OCT molds for storage at −80 C. Thirty-micron sections were cryosectioned (Leica CM1950) and stored in cryoprotectant at −20 C.

#### qPCR

RNA was isolated using the TRIzol method and secondary clean-up was performed using a column and integrated DNase digestion method (Zymo Research, #R1017) following manufacturer protocol. Isolated RNA was evaluated by spectrophotometry (NanoDrop, Thermo Scientific) and all samples with A260/280 ratios between 1.8 and 2.0, and A260/230 ratios between 2.0 and 2.2 were included. BioRad iScript kit (#1708890) was used to generate cDNA which was stored at −20 C. One tenth of the volume of cDNA reaction was used downstream with iTaQ Universal SYBR supermix (BioRad, #1725120) according to the manufacturer protocol. Reactions were performed in triplicate and a final, averaged Cq value used for analysis. Normalization occurred first to the reference gene β-actin and the relative changes in expression levels compared to controls were calculated according to the 2^−ΔΔct^method (Livak and Schmittgen 2001). An inter-plate control was run across all plates to allow use of the CFX Maestro Gene study function for analysis (BioRad, v1.1). One statistical outlier was excluded in the water group for all genes inspected except for*Gad1*. One statistical outlier was excluded from the ethanol group for *Gabra4*, and two outliers each were excluded from the ethanol group for *Gabrd* and *Slc12a5*.

#### Immunohistochemistry

Tissue was removed from cryoprotectant, and slices containing PVN were selected. Slices were incubated in 0.3% citrate buffer (45 min at 65 C and 30 min at RT) for antigen retrieval. To block nonspecific signal, slices were incubated in 10% normal goat serum (NGS) for one hour and incubated overnight in primary antibody (rabbit anti-cFOS Cell Signal, #2250S; rabbit anti-KCC2 Cell Signal, #94725; 1:1000 in 1% NGS 0.3% PBST) at 4 C. For cFOS, a two-hour incubation at RT with a biotinylated anti-rabbit antibody (Vector Labs, #ZF0920, 1:1000 in 1% NGS 0.3% PBST) preceded a final incubation with a Streptavidin-conjugated Alexa-Fluor 488 (Thermo Fisher, #S11223, 1:1000 in 1% NGS 0.3% PBST), both done at RT for two hours. For KCC2, slices were incubated for two hours at RT with an Alexa-Fluor 488 conjugated anti-rabbit secondary (Thermo Fisher, #A-11008). Slices were stored in PBS until mounting on charged slides (Fisher Scientific, #22037246) and cover-slipped with a hardset mounting medium with DAPI (Vector Laboratories, #H-1500).

#### Confocal imaging and analysis

Slides were imaged on a Leica SP8 confocal microscope with 20X objective using OPSL 488 (2% power for cFOS, 2.5% power for KCC2) and Diode 405 (1% power) lasers at 1024 × 1024 resolution with 400 speed bidirectionally, in five-micron steps. For KCC2, 4-line averages and 2 frame accumulations were used. LAS-X directed navigator was used to acquire, tile, and merge region of interests. For analysis of cFos immunoreactivity, merged images were imported into QuPath (Bankhead 2017). Coronal sections of interest were defined based on highest CRH expression as seen in Allen Brain ISH expression atlas (experiment #292). ABBA was then used to align and register anatomically appropriate slices-based Allen Brain Atlas Mouse Coronal Atlas in FIJI (v0.8.0). Registered slices were background corrected in the 488 channel in QuPath and registered PVN annotation was selected in each section in both hemispheres for the cell detect function. Cells that co-expressed cFOS and DAPI were summed inboth hemisphere of each section, and replicated across three bilateral sections per animal. For analysis of KCC2 immunoreactivity, region of interest selection was performed with FIJI and integrated densities were measured in each hemisphere of each section and replicated across three sections per animal.

#### Statistics

Data were analyzed using GraphPad Prism (v10.1.0) and JASP (v0.19.0). ROUT tests in Prism were used to identify and remove statistical outliers at a false discovery rate (Q) of 1% prior to conducting analyses in JASP. Normality of the data was assessed using the Shapiro-Wilk test.

Mixed two-way ANOVAs were used to examine the effects of sex (male or female) and day on ethanol intake across 14 days of access. Two-way ANOVAs were also used to assess the effects of adolescent drinking history (ethanol or water) and sex on behavioral reactivity in the forced swim stress and social defeat stress assays, endocrine reactivity following social defeat stress, and gene expression levels measured by qPCR.

A mixed-design three-way ANOVA was used to evaluate changes in corticosterone levels over time following forced swim stress. When the assumption of homogeneity of variance was violated (as assessed by Levene’s test), nonparametric Kruskal-Wallis tests were applied. Greenhouse-Geisser corrections were used when the assumption of sphericity was violated, and adjusted degrees of freedom were reported accordingly.

Planned contrasts were conducted within each sex to evaluate the effects of adolescent drinking history, with Bonferroni correction applied to control for multiple comparisons.

For immunohistochemical analyses of cFOS and KCC2, linear mixed models (LMMs) were conducted using restricted maximum likelihood (REML) estimation. To account for repeated measures across brain sections, individual animal ID was included as a random effect.

Effect sizes (Cohen’s d, η² or Rank ε^2^ as appropriate) are reported for all findings significant at an α = 0.05 threshold.

## Results

### Adolescent male and female C57BL/6J mice consume similar amounts of ethanol and display similar impact of zeitgeber time on binge consumption pattern over days

A mixed two-way ANOVA was used to assess changes in the total ethanol (g/kg) consumed each day between sex (male *n* = 24; female *n* = 21; Fig. 1B). There was a significant effect of day on the amount of ethanol consumed (F_7.174, 308.492_=3.07, *p* = 0.004, η^2^ = 0.05). Post-hoc analyses clarified that mice ramped up their drinking by the third day of access (day 1 vs. day 3=−4.16, *p* = 0.01, d=−0.99) but tapered and stabilized consumption such that ethanol intake on day 14 was not significantly greater than intakes on the first day (male M ± SEM = 4.52 ± 0.29, female M ± SEM = 5.12 ± 0.3 g/kg/3hrs, day 1 vs. day 14 (t=−2.45, *p* = 1.00, Fig. 1B)). There was no significant interaction of sex and day (F_7.174, 308.492_= 0.86, *p* = 0.54), nor was there a significant main effect of sex (F_1,43_=3.06, *p* = 0.09). These analyses thus supported similar alcohol intakes across sex and stable daily consumption across most of the two weeks of access for both males and females. While we did not employ blood ethanol concentration measures, prior work has shown that similar drinking levels under this DID-MSA paradigm are sufficient to result in blood alcohol levels that are binge-like and induce behavioral intoxication.

To examine sex differences in the circadian pattern of intake across the two weeks, the amount alcohol consumed during each of the three hourly access sessions were compared using mixed three-way ANOVAs with day (1, 7, and 14), time (ZT12, ZT15, ZT18), and sex (male vs. female) as factors. For both males and females, there was no statistically significant change in the trends at each session (first binge session at ZT12, second binge session at ZT15, third binge session at ZT18) across days 1, 7, and 14 (F_4,172_ =0.68, *p* = 0.61; Fig. 1C). There was a significant interaction of drinking sessions and days (F_4,172_=4.23, *p* = 0.003, η^2^ = 0.029) as well as a significant main effect of time (F_2, 172_=20.4, *p* < 0.001, η^2^ = 0.09) and a significant main effect of day (F_2, 172_=4.254, *p* = 0.017, η^2^ = 0.018). Post hoc tests showed that by day 7, alcohol consumption during the third access period (ZT18) was significantly lower than during earlier sessions (ZT12 and ZT15), particularly on Day 7 (ZT12 vs. ZT18: t = 5.34, *p* < 0.0001, d = 1.21; ZT15 vs. ZT18: t = 5.44, *p* < 0.001, d = 1.15) and Day 14 (ZT15 vs. ZT18: t = 4.26, *p* = 0.004, d = 0.59). The only exception was on Day 14, where ZT12 still showed significantly higher intake than ZT18 (t = 3.19, *p* = 0.0097, d = 0.06).

### Adult females with a history of adolescent binge drinking show increased immobility in response to forced swim stress

**Fig. 2:**
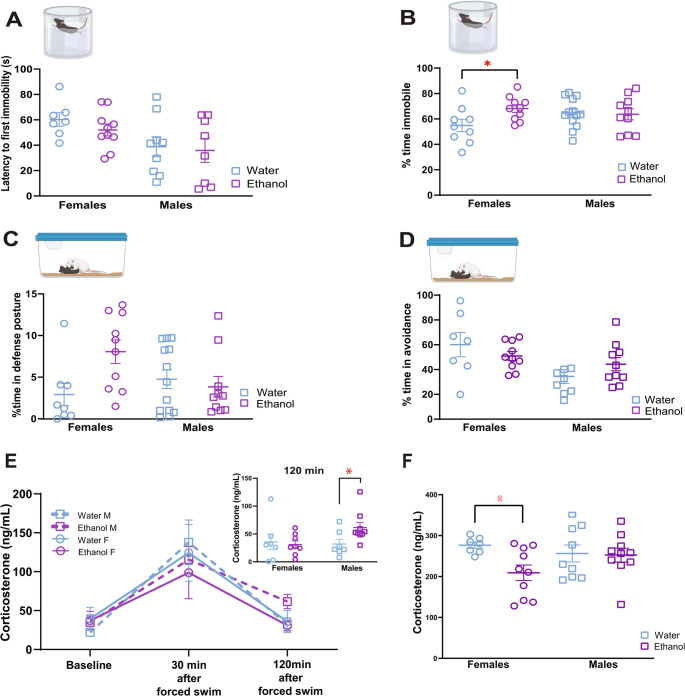
Adolescent binge drinkers show sex and stressor specific impact of adolescent alcohol in their behavioral and CORT response to stressors. (**A**) Neither sex shows an impact of adolescent binge drinking in their latency to the first bout of immobility in the forced swim stress. (**B**) Females with a history of adolescent binge drinking show a higher percentage of time immobile in response to a forced swim stressor (t_46_ = 2.07, *p* = 0.04, d = 0.87). (**C**) Neither sex shows an impact of adolescent binge drinking on percent time spent in defense posture during social defeat stress. (**D**) Neither sex shows an impact of adolescent binge drinking on percent time spent in avoidance behavior during social defeat stress. (**E**) For the CORT response to forced swim stress, only males with an adolescent binge drinking history fail to return to baseline 120 min after forced swim stress (t_27_=−2.14, *p* = 0.04, d=−0.56). (**F**) For the CORT response to social defeat stress, only females with an adolescent binge drinking history show significantly reduced CORT response 30 min after SDS (U = 11, *p* = 0.02, d=−0.69). * = *p* < 0.05’s; n’s = 10 per sex for ethanol drinkers and 5–9 per sex for water controls

Next, we assessed the impact of adolescent binge drinking on later adult behavioral responses to two stressors (n’s = 8–14 per sex, per treatment group). To determine whether a history of adolescent drinking would impact behavioral response to a general psychological stressor, we evaluated the latency to the first bout of immobility (Fig. 2A) and the percent of time immobile in the forced swim stress (Fig. 2B). A two-way ANOVA (adolescent binge drinking history X sex) of the first bout of immobility during the forced swim stress supported a significant main effect of sex (F_1,32_ = 5.49, *p* = 0.03, η^2^ = 0.14). This analysis did not support a significant interaction of sex and adolescent alcohol history (F_1,32_ = 0.55, *p* = 0.46).

A two-way ANOVA of the percent of time spent immobile during the forced swim stress supported an interaction of adolescent binge drinking history and sex (F1,46 = 4.1, *p* = 0.049, η^2^ = 0.079). Planned contrasts did not show a significant difference in time spent immobile (t_46_=−0.74, *p* = 0.46) between adolescent binge drinkers (M ± SEM = 62.24 ± 3.35) and water drinkers (M ± SEM = 64.68 ± 3.23). For females, a history of adolescent binge drinking (M ± SEM = 64.94 ± 2.86) was associated with a significant increase in the time spent immobile in the forced swim stress task in adulthood (t_46_ = 2.07, *p* = 0.04, d = 0.87, Fig. 2B), when compared to water controls (M ± SEM = 54.46 ± 4.37).

To determine whether a history of adolescent drinking would alter behavioral response to a social stressor, the percent of time spent in defense posture (Fig. 2C) and avoidance (Fig. 2D) were quantified during the social defeat stress task. Kruskal Wallis analysis of defense behavior showed no effect of sex (H = 2.84, *p* = 0.09, Rank ε^2^ = 0.06) or adolescent binge drinking history on percent time spent in defense posture (H = 0.75, *p* = 0.39). Mann Whitney U tests as planned contrasts showed no effect of adolescent history on defense posture within either sex (males U = 87, *p* = 0.8; females U = 84, *p* = 0.26).

Kruskal Wallis analysis of avoidance behavior showed a significant effect of sex (H = 8.24, *p* = 0.004, Rank ε^2^ = 0.24), as females presented avoidance behavior for a greater portion of the engagement than males overall. However, adolescent binge drinking history did not impact the percent time spent engaging in avoidance behavior (H = 1.14, *p* = 0.29). Mann Whitney U tests as planned contrasts showed no effect of adolescent history on avoidance posture within either sex (males U = 61, *p* = 0.07, females U = 26, *p* = 0.42).

### Adult females with a history of adolescent binge drinking show reduced CORT reactivity to social defeat stress and males with an adolescent drinking history show delayed recovery of CORT levels following forced swim stressor

To assess the impact of adolescent drinking on the neuroendocrine stress response, CORT levels were measured at baseline, 30 min after forced swim (stress reactivity), and 120 min after (stress recovery) in each sex and treatment group (*n* = 5–10/group). A repeated measures ANOVA, corrected with Greenhouse- Geisser estimates, found no significant three-way interaction between adolescent drinking history, sex, and CORT levels (F_1.24, 36.25_ = 0.17, *p* = 0.17; Fig. 2E).

Planned contrasts showed no effect of adolescent alcohol history on baseline (t₂₇ = 0.33, *p* = 0.74), stress reactivity (t₂₇ = 0.61, *p* = 0.55), or recovery CORT levels in females (t₂₇ = 0.35, *p* = 0.73; Fig. 2E). In males, adolescent alcohol history had no effect on baseline or stress reactivity CORT (t₂₇ = −0.79 and 0.55, respectively; both *p* > 0.4), but significantly elevated recovery CORT levels at 120 min post-stress (t₂₇ = 2.14, *p* = 0.04, *d* = −0.56). Males with a history of adolescent binge drinking showed recovery CORT levels 90% higher (61.61 ± 9.17) than water controls (32.12 ± 8.51).

To determine whether a history of adolescent drinking would impact the endocrine response to a psychosocial stressor, blood was sampled 30 min after exposure to a sex-matched trained aggressive CFW mouse. Kruskal Wallis showed no impact of adolescent binge drinking history (H = 2.96, *p* = 0.09) or sex (H = 0.18, *p* = 0.67) on CORT levels 30 min following social stress.

For females, Mann Whitney U tests as planned contrasts supported an impact on this measure for females (U = 11, *p* = 0.02, d=−0.69, Fig. 2F), as CORT levels following social defeat stress was lower in females with a history of adolescent binge drinking (M ± SEM = 209.08 ± 18.97) compared to water controls (M ± SEM = 276.5 ± 7.43). For males, this analysis did not support an effect of adolescent history on CORT reactivity to social stress (U = 48, *p* = 0.84; Fig. 2F).

### Adult males with a history of adolescent binge drinking show overexpression of KCC2 (SLC12A5) in PVN-enriched tissue

**Fig. 3:**
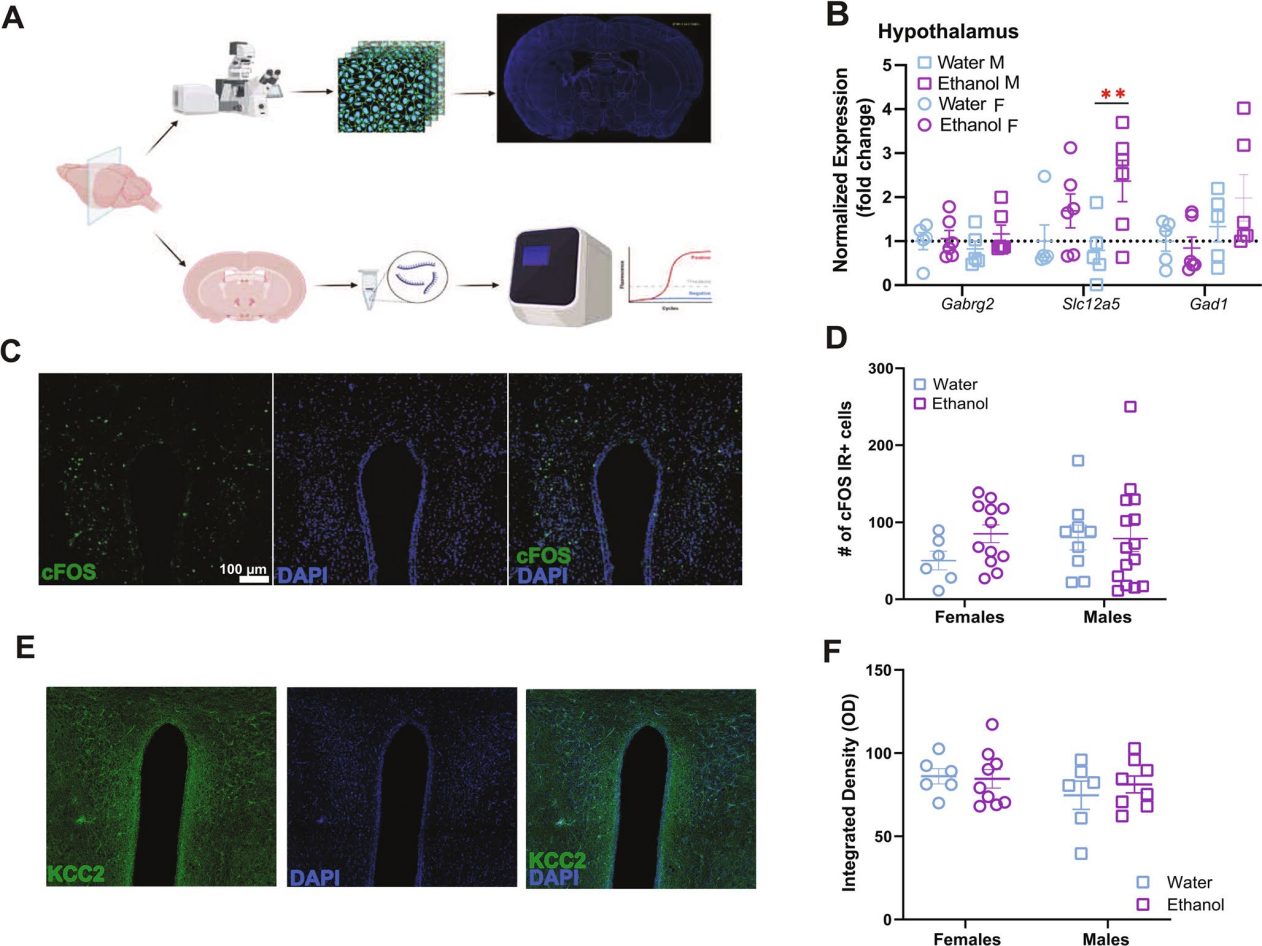
Adolescent binge drinking history increases *Slc12a5* expression in the hypothalamus enriched tissue only in males. (**A**) Experimental timeline. (**B**) Only males with a history of adolescent binge drinking show elevated expression of *Slc12a5* (KCC2*)* in PVN-hypothalamic enriched tissue (ethanol vs. water; t_18_ = 3.56, *p* = 0.002, d = 2.16) sampled during adulthood. (**C**) Representative confocal images for cFOS immunoreactivity. (**D**) cFOS immunoreactivity in the PVN of the hypothalamus is not impacted by a history of adolescent binge drinking in either sex. (**E**) Representative confocal images for KCC2 immunoreactivity. (**F**) Representative confocal images for KCC2 analysis. (**G**) KCC2 expression in the PVN is not impacted by adolescent binge drinking history or sex

We investigated whether the endocrine and behavioral effects of adolescent exposure were due to changes in inhibitory control at the level of the hypothalamus. We isolated RNA from hypothalamic PVN- enriched tissue of both males and females (n’s = 5–6 per sex per treatment group) with a history of adolescent binge drinking and performed qPCR for genes relevant to GABAergic signaling: *Gabrg2*, *Gad1*, and *Slc12a5* (Fig. 3B). Two-way ANOVAs were performed to assess the impact of adolescent binge drinking and sex on the expression of each gene. Transcript levels for *Gabrg2*, *Gad1* remained consistent across treatment group and sex. There was a marginal interaction between the effects of sex and adolescent binge drinking on expression of KCC2’s gene *Slc12a5* in the PVN (F_1,18_ =4.29, *p* = 0.05). Planned contrasts revealed a significant increase (t_18_ = 3.56, *p* = 0.002, d = 2.16, Fig. 3B) in the expression of *Slc12a5* transcript (KCC2) in PVN-enriched tissue isolated from adult males with a history of adolescent binge drinking (M ± SEM = 2.37 ± 0.47) when compared to water only controls (M ± SEM = 0.79 ± 0.31). Females did not show this effect of drinking history.

To determine whether a history of adolescent drinking would alter reactivity of cells in the paraventricular region of the hypothalamus, we counted cFOS reactive neurons in brains harvested 30 min following social defeat stress (Fig. 3D). Linear mixed model (LMM) was conducted to assess impact of sex and ethanol treatment on this measure with individual differences as random effects. The model was estimated using restricted maximum likelihood (REML) method. This analysis did not support a significant impact of binge drinking history or sex on the number of cFOS positive cells in the PVN (Sex, F_1,11.99_=0.05, *p* = 0.82; Treatment, F_1,11.5_=0.50, *p* = 0.49).

To test whether the changes in *Slc12a5* transcripts translate into protein levels, we performed KCC2 immunohistochemistry in sections containing PVN that are at similar anatomically registered levels as were used for the cFOS quantification (Fig. 3F). Linear mixed model analysis with individual differences as random effects did not support a significant interaction of adolescent binge drinking history and sex on KCC2 immunoreactivity measured by integrated density in the PVN (Sex, F_1,7.26_=0.77, *p* = 0.407; Treatment, F_1,7.43_=0.097, *p* = 0.76).

## Discussion

The current study utilized a pair-housed binge drinking model to evaluate sex differences in the relationship among adolescent binge drinking, adult neuropsychoendocrine response to varied stressor modalities, and inhibitory signaling in the hypothalamus. The findings presented confirm that adolescent binge drinking leads to enduring sex-specific deficits in stress signaling in adulthood and show for the first time that adolescent pair-housed binge drinking leads to sex-specific changes in *Slc12a5* expression in the hypothalamus. These results extend our current understanding of the effects of adolescent binge drinking on maturation of the stress axis. The findings most importantly highlight that neither sex is resilient to the effects of adolescent binge drinking: the sexually diergic expression of psychoneuroendocrine dysfunction following adolescent binge is context dependent.

### Adult male mice with a history of adolescent binge drinking in a pair-housed drinking model show no changes in behavioral reactivity to either social nor non-social stress

Adult males with a history of adolescent binge drinking in our study did not show altered behavioral responses to either a forced swim or a social stressor. This aspect of our work did not replicate previous findings in mice showing an effect of adolescent intermittent ethanol drinking on behavioral reactivity to a forced swim stressor (Khan et al. 2023; Lee et al. 2017). A significant difference between the studies includes our use of social housing during adolescent alcohol exposure. Stress associated with isolation housing is known to synergize with alcohol to uniquely impact adult stress and anxiety in rodent models (Lopez et al. 2011; Lopez and Laber 2015). We believe the use of a pair-housed drinking paradigm helps to isolate the effect of adolescent binge drinking history on the developing stress system. Additionally, we did not employ a sub-chronic stressor prior to our stress test which may importantly be needed to unmask the impact of adolescent binge drinking. Indeed, male rats with a history of adolescent intermittent ethanol do not show an impact of alcohol in forced swim or sucrose preference unless treated with a sub-chronic stressor beforehand (Healey et al. 2023)but show no change in social interaction during abstinence in adulthood (Van Hees et al. 2022).

Ethanol access was removed until adulthood, thus our mice experienced not just the effect of adolescent alcohol exposure, but also the impact of changes associated with withdrawal itself can have on a developing system. Further, abstinence is associated with recovery of some alcohol associated pathologies. Indeed, in rats, early adolescent intermittent ethanol results in increased anxiety-like behaviors that diminish over time during protracted abstinence (Matthews et al. 2019). Thus, the three weeks of abstinence following their adolescent drinking history may have permitted recovery of any deficits in early stress reactivity for our males.

Our results did not support an effect of adolescent ethanol drinking on behavioral responses to social defeat stress in the two behaviors assessed for either sex. Social defeat stress is an etiologically relevant stressor with many subtle behavioral indicators, and it is possible that our analysis which measured the display of defensive or avoidance behaviors, was not granular enough. As our social stressor came following the non-social stress experience, where males showed delayed recovery of a CORT response, this was surprising, as higher CORT levels during a defeat engagement is inversely correlated to fighting and guarding behaviors (Walker et al. 2009). However, the time lapse between the two behavioral experiences may have been sufficient for the CORT levels to return to baseline by the time social defeat stress protocol was initiated, as there was a 3-hour waiting period between stressors.

### Adult male C57BL/6J mice with a history of adolescent binge drinking show impaired recovery of CORT following forced swim stress

Adult male mice with a history of adolescent pair-housed binge drinking showed significantly elevated CORT levels two hours following exposure to a swim stressor, when CORT levels should have returned to baseline (Pastor et al. 2012). During peri-adolescence in males, CORT reactivity to stressors is similar to that seen in adult, but termination of this endocrine response is often delayed in adolescent males. This difference is more pronounced with certain stressors such as foot shock compared to others like restraint. This is thought to be due to the immature nature of the negative feedback mechanism in peri-adolescence, as clearing rates of CORT in males and females are similar (McCormick and Mathews 2007, 2010). Our results of prolonged CORT response in binge drinker males are reminiscent of this feature of the peri-adolescent HPA axis, suggesting that this too may be subject to the ‘lock in’ phenomenon of arrested development following early alcohol exposure (Crews et al. 2019).

In our study, males with a history of early adolescent binge drinking showed no difference in basal CORT or stress reactivity CORT following exposure to swim stress when compared to water drinking controls. Our results are in line with the literature that adolescent intermittent ethanol may not impact early CORT reactivity following a single session of non-social stress (Varlinskaya et al. 2017). However, findings in rats highlight a unique susceptibility to repeated stress, as adult rats with a history of adolescent binge drinking fail to habituate to repeated restraint stress (Varlinskaya et al. 2017). It is possible that the delayed endocrine response to an acute stress noted for our mice could underlie the development of aberrant HPA axis activity following multiple stress exposures. Still, our data suggest that this sex-specific effect of adolescent binge drinking noted here is relevant to termination of HPA axis response, but not activation or reactivity. Further, as these males showed no such difference in their endocrine reactivity to a social stressor, our findings support previous work that stressor modality is important to the feedback regulation and neurocircuitry recruited to terminate the HPA response.

### Females with a history of adolescent binge drinking show increased immobility in a forced swim stress task

A large body of work emphasizes unique male-specific vulnerability to the effects of early adolescent binge drinking on later reactivity to stress in adulthood in rodent models. In rats, adolescent intermittent ethanol induces increased preference for saccharin in females, but no change in immobility while engaged in a forced swim task. Investigations of impact of stress on anxiety like measures following adolescent alcohol did not uncover any changes to the anxiety like behaviors in female rats following sub-chronic stress (Healey et al. 2023). In a similar study, females with history of adolescent intermittent ethanol only displayed abnormal social interaction, social preference, or locomotor activity from their water control counterparts following a prior stress (Varlinskaya et al. 2017). Even though we did not employ a stressor before our tests, we did see increased immobility time in forced swim stress in the adolescent binger females in our study. The difference that might contribute to the discrepancy between our findings and aforementioned reports could be the mode of ethanol administration, where the cited reports employed gavage mode of administration while we employed voluntary binge drinking. Therefore, although the interaction of adolescent alcohol exposure and stress could explain the contrast between the initial findings and other work, the use of pair-housed binge drinking in our study removes this potential effect.

We believe the withdrawal period embedded in our design as mice waited to be tested in adulthood, afforded an incubation period required for the revelation of the stress related findings. Indeed, a recent report showing the significance of this incubation period in the display of abnormal anxiety and stress related behaviors following adolescent binge drinking found immobility in the forced swim stress following adolescent drinking at protracted (40 days), but not early (72 h), withdrawal (Van Hees et al. 2022).

We did not find any impact of adolescent alcohol exposure on females’ presentation of defense or avoidance behaviors when faced with a trained aggressor. Although prior studies have examined how ethanol and chronic social defeat stress affect drinking and stress measures in rodents (Boutros et al. 2016; Newman et al. 2018; Reguilón et al. 2023), to our knowledge, this is the first study to use social defeat stress as an acute stressor during withdrawal from adolescent pair-housed binge drinking.

### Adult female mice with a history of adolescent binge drinking show endocrine hypo-reactivity to a social stressor

Unlike males in our study, adult female mice with a history of adolescent pair-housed binge drinking show altered neuroendocrine responses to the social stressor. Female rats show a higher CORT response to swim stress but not males (Vecchiarelli et al. 2022). In female C57BL/6J mice, chronic stress is shown to result in lower-than-normal recovery CORT levels 90 min following forced swim stress, but does not impact the behavioral response to the stressor (Varlinskaya et al. 2017). Our females did not show an impact of adolescent binge drinking history on CORT response to forced swim stress. Unlike males in our study, adult female mice with a history of adolescent pair-housed binge drinking exhibited altered neuroendocrine responses to social stress. Female rats exhibit a higher corticosterone (CORT) response to forced swim stress, an effect not seen in males (Vecchiarelli et al. 2022). In female C57BL/6J mice, chronic stress has been shown to lower recovery CORT levels 90 min after forced swim stress without altering behavioral responses (Varlinskaya et al. 2017). Our female mice did not show altered CORT responses to forced swim stress following adolescent binge drinking. However, they did exhibit significantly reduced CORT levels following social stress compared to water-drinking controls. Prior to pubertal maturation, females show a higher peak CORT in response to stress, but this peak occurs later than in adults. Because adolescent females exhibit a slower rise in CORT, their stress response may appear hyporeactive when adult levels peak, but eventually surpasses them (Burke and Miczek 2014; Klein and Romeo 2013; McCormick and Mathews 2010). The recovery timeline of the CORT response is similar in both adolescents and adults with CORT levels returning to baseline around 90–120 min. The lack of a middle sampling point could therefore have prevented us from discovering the point where the CORT levels for the drinkers surpass the non-drinkers. This hyporeactivity we observed could reflect an immature HPA axis response to stressors, suggesting that, as in males, a ‘lock-in’ of immature stress responsivity following early adolescent alcohol exposure (Crews et al. 2019) may play a role in females as well.

It should also be noted that our stress paradigm could be classified as a modified two-hit approach, since the two stress tests were separated by a three-hour interval. In male mice, restraint stress has been shown to increase anxiety-related behaviors, like more time spent in the closed arms of the elevated plus maze and near the walls in an open field maze, within 30 min after the stressor. These measures tend to normalize and approach those of unstressed animals as the time between stress exposure and behavioral testing extends to 12–24 h (Chen et al. 2024; Liu et al. 2023). Therefore, the behavioral responses our animals presented in face of social defeat stress may have been affected by the prior forced swim stress. However, the 3-hour difference should have been enough to normalize the neuroendocrine system as the shutdown of the CORT response occurs within 90–120 min. Regardless, since all mice were exposed to both stressors in the exact same timeframe, the differences we see can be attributed to alcohol’s impact on the regulation of the stress systems.

We did not find any changes in the tested GABAergic transcript levels in the hypothalamus of our females with a history of adolescent binge drinking. It is possible then that these females, as in prior work, may show unique changes to excitatory glutamatergic signaling systems in this region instead. Indeed, it was recently shown that adult female mice with a history of binge drinking show a blunted acute neuroendocrine response to a mild homecage stressor and enhanced glutamatergic signaling in PVN CRH neurons during acute withdrawal (Neira et al. 2023). The blunted CORT response to social stress that we see in females with a history of adolescent drinking may similarly reflect an impact of alcohol broadly on the initial engagement of the hypothalamus to stress. Moreover, the impact of adolescent drinking in our experiment did not extend to neuroendocrine reactivity to the forced swim stress, highlighting the importance of stressor-modality in these deficits.

### Males with a history of adolescent binge drinking show increased expression of KCC2 in PVN-enriched tissue

Our investigation of expression of genes relevant to GABAergic signaling in the hypothalamus enriched tissue showed that only males with a history of adolescent binge drinking had greater KCC2 transcript (*Slc12a5*). Expression of the gene responsible for the main subunit protein, *Gabrg2*, expressed by synaptic GABA_A_ receptors, or the gene responsible for the enzyme GAD67 that catalyzes the majority of brain GABA, was not affected. Previous work investigating the impacts of adolescent binge drinking on male mice did not detect any changes to *Slc12a5*(Zou et al. 2014). However, that study did not investigate the effects of withdrawal. It is possible that the changes in*Slc12a5* expression could require an incubation period or some period of withdrawal.

Interestingly, this change was absent in females. In fact, while females showed the majority of the behavioral and endocrine deficits associated with a history of adolescent binge drinking in our study, yet we did not detect any significant change in the expression *Slc12a5*, *Gabrg2*, or *Gad1*. We focused on GABAergic signaling given the major contribution to local and surrounding inputs to the paraventricular region of the hypothalamus and significance to regulation of CRH activity in the region (Rigney et al. 2020). KCC2 expression is crucial in maintaining the Cl- levels in neurons to ensure GABA acts as inhibitory (Sarkar et al. 2011)but its downregulation plays an important role in the shift in shunting inhibition necessary to permit enhanced firing of CRH neurons in the hypothalamus and proper reactivity to stress (Melón and Maguire 2016).

To our knowledge, this is the first study to demonstrate an elevation of Slc12a5 gene expression in the hypothalamus of males following adolescent binge drinking. The functional implications of this increase require further investigation. However, given that its protein product, KCC2, plays a critical role in maintaining low intracellular chloride levels and supporting inhibitory GABAergic signaling, changes in Slc12a5 expression may indicate altered GABAergic regulation within the hypothalamus (Melón and Maguire 2016). This is particularly noteworthy because KCC2 downregulation in the hypothalamus is known to relieve shunting inhibition of CRH neurons, a mechanism essential for proper stress responsiveness (Melón et al. 2018; Motta and Canteras 2015). If increased Slc12a5 expression leads to greater levels of functional KCC2, it could enhance chloride efflux, lowering intracellular Cl⁻ and strengthening the inhibitory effects of GABA. However, several caveats limit the interpretation of this finding. Elevated mRNA levels do not necessarily translate to increased functional, phosphorylated, and dimerized KCC2 protein. The rise in transcript levels may reflect a compensatory response to accelerated protein turnover, misfolding, or other mechanisms that impair KCC2 function, potentially nullifying the physiological effect of the increase.

Additionally, the balance between NKCC1 and KCC2 must be considered. Although NKCC1 expression typically decreases as neurons mature, it remains active in some mature neurons. Thus, concurrent upregulation of NKCC1 could offset the impact of elevated KCC2 by promoting chloride influx, potentially maintaining—or even increasing—intracellular Cl⁻ levels. Future studies should evaluate this interplay to determine whether increased *Slc12a5* expression truly alters inhibitory tone in the hypothalamus.

On the other hand, the timing of tissue collection in our experimental design critically shapes how these results should be interpreted. Samples collected 30 min after the second stressor primarily reflect acute transcriptional responses, such as *Slc12a5* upregulation, since mRNA synthesis can occur within minutes. In contrast, KCC2 protein levels are governed by slower processes including translation, trafficking and post-translational modifications, that may require several hours to manifest. Therefore, the absence of measurable KCC2 changes at 30 min likely reflects timing rather than a true lack of biological impact. The increased *Slc12a5* mRNA observed in males with a history of adolescent drinking might have produced detectable protein changes if examined later. Furthermore, because the forced swim and social defeat stressors were separated by three hours, the mRNA differences we detected represent cumulative rather than immediate effects of both stressors. Together, these findings underscore a temporal and mechanistic decoupling between transcription and protein response, shaped by both sex and stress modality. Only males exhibited elevated *Slc12a5* transcripts following social stress, whereas neither sex showed corresponding protein alterations 3.5 h post-stress, suggesting possibility for delayed or posttranslational regulation of KCC2 protein in males.

Importantly, our finding aligns with clinical evidence linking adolescent alcohol exposure to an increased risk of stress-related disorders and alcohol use disorder (AUD), even in individuals who abstain in adulthood. Recent work has shown that CRH-expressing neurons in the paraventricular nucleus (PVN) are significantly affected by intermittent alcohol exposure in female mice (Neira et al. 2023). If such alterations occur regardless of developmental timing, our findings may complement that study. The elevated KCC2 observed in males may represent a compensatory response to increased glutamatergic signaling previously reported in both adult males and females following intermittent alcohol exposure. In this context, enhanced KCC2 expression could serve a protective role by dampening excitability in CRH neurons, thereby preventing the hyperexcitable CRH profile observed in females.

### Neither sex shows an impact of adolescent binge drinking in number of cFOS immunoreactive cells in the PVN of the hypothalamus 30 min after social stress in adulthood

In mice, cFOS signals are validated within the PVN for CRH cell activity to stressors (Newman et al. 2018; Walker et al. 2019). In adult female mice, social defeat stress leads to increase in cFOS positive cells in PVN (Newman et al. 2019). Both repeated stress, and adult alcohol combined with repeated stress increase cFOS reactivity in the PVN of adult male and female mice (Clark et al. 2023). During acute withdrawal from chronic intermittent ethanol total number of cFOS positive cells in the PVN decreased in females but remained unchanged in males (Neira et al. 2023). Following adolescent alcohol, in male rats number of neurons with cFOS colocalization with CRH was increased (Allen et al. 2016). However, in male mice during early withdrawal from chronic ethanol (72 h) results in increased number of cFOS positive cells, the numbers normalize as the withdrawal lengthens (Smith et al. 2020). We expected to see a change in the number of cFOS immunoreactive cells in the PVN of males and females with a history of adolescent binge drinking. We also expected to see a change in number of KCC2 immunoreactive neurons especially given the change in*Slc12a5*expression we observed for males. Instead, we found no significant impact of history of adolescent binge drinking on cFOS or KCC2 immunoreactivity in the PVN following social stressor in either sex. There may be several reasons behind this discrepancy between our results. Firstly, our cFOS analysis was performed without CRH co-staining, and our counts may include vasopressin cells which are also activated following social defeat stress (Rigney et al. 2020; Melón et al. 2018). Additionally, while cFOS expression starts around 30 min following neuronal activity, it reaches its peak later. This is one of the constraints of our design. Therefore, we may not have detected the full scope of changes in cFOS levels at peak. For a more definitive conclusion either a later timepoint or an mRNA biochemical assay would need to be performed. Secondly, although our KCC2 mRNA analysis was performed with isolated tissue enriched with PVN of the hypothalamus, this was a gross dissection and thus the changes we found may also reflect RNA isolated from neighboring hypothalamic nuclei. Thirdly, an increase in mRNA does not always correlate to active protein form, especially since KCC2 has a fast turnover rate, and phosphorylation of the protein plays a significant role in its activity. Thus, further biochemical assays need to be performed to reach a more definitive conclusion. Lastly, we appreciate that even if these changes in transcription of*Slc12a5* following adolescent binge drinking for males translated to aberrant upregulation of KCC2 activity in the PVN, changes in the glutamatergic system or other regulatory systems that project to those cells may stabilize activity such that reactivity to a singular social stressor remained unchanged.

## Conclusions

Taken together our results support that no single sex is resilient to the effects of early adolescent binge drinking, even in a pair-housed drinking paradigm. We show that, instead, the stressor type (social vs. non-social) and aspect of the stress axis impacted (reactivity vs. recovery) is sex dependent. Males with a history of adolescent drinking showed no impact of this drinking history on the behaviors tested following social or non-social stress tests, but showed impaired recovery of CORT levels following stress, enhanced expression of KCC2 in the hypothalamus and lasting neuroendocrine effects. Females, on the other hand, showed persistent behavioral deficits in response to both stress modalities, yet showed endocrine hyporeactivity following only social stress. This difference suggests that the point of impact of adolescent binge drinking on the developing hypothalamic stress circuitry may be different for males and females. Further research is needed to fully understand the implications of these changes however they may underlie the increased risk of developing AUD or stress disorders in adulthood following adolescent binge drinking.

## Data Availability

All data presented herein can be made available by contacting the corresponding author.
